# Employee Stock Ownership Plans and Corporate Environmental Performance: Evidence from China

**DOI:** 10.3390/ijerph20021467

**Published:** 2023-01-13

**Authors:** Hongfeng Sun, Chang Liu

**Affiliations:** 1 School of Accounting, Zhongnan University of Economics and Law, Wuhan 430073, China; 2 School of Economics and Management, Shihezi University, Shihezi 832003, China

**Keywords:** managerial incentives, employee stock ownership plans, environmental performance, productivity, green technology

## Abstract

In the context of corporate sustainability, studies on the role that managerial incentives play in improving corporate environmental performance have so far focused on incentives provided either to executives and senior managers or to plant managers. However, few studies have considered the role of employee incentives. Drawing on the opportunity provided by the China Securities Regulatory Commission in restarting employee stock ownership plans (ESOPs) in 2014, this paper investigates the impact of employee incentives on environmental performance of high-polluting enterprises. The results indicate that ESOPs are significantly positively related to corporate environmental performance. The positive effect is particularly pronounced in subsamples with weak free-riding problems, high human capital quality, and non-state-owned enterprises (non-SOEs). Further analysis reveals that ESOPs improve corporate environmental performance through enhancing productivity and green technology. Overall, this paper reveals the micro-mechanisms behind the actual effects of employee incentives on corporate environmental management, thus providing timely implications for high-polluting enterprises to improve environmental performance.

## 1. Introduction

The activities of high-polluting enterprises are the primary source of pollution [1]. In the context of sustainable development, improving the environmental performance of high-polluting enterprises has received increasing attention from various stakeholders, such as investors, customers, governments, and the public [2]. According to the resource-based theory, human capital plays a crucial role in the environmental management activities and sustainable development of enterprises [3]. Hence, fully developing the potential of human capital and mobilizing the enthusiasm of human capital in corporate environmental strategy has become an important driving force for enterprises to improve environmental performance [4]. Around this perspective, the existing research literature mainly examines the impact of managerial incentives on corporate environmental performance. However, many of the studies have so far focused on incentives provided either to executives and senior managers or to plant managers [5,6,7]. There is a lack of empirical research that examines the role of employee incentives in corporate environmental performance [8].

In fact, many scholars have realized the significance of employees in corporate environmental management [9,10]. For instance, Wehrmeyer and Parker (1995) [11] asserted that “if a company is to adopt an environmentally aware approach to its activities, the employees are the key to its success or failure”. Zhu et al. (2021) [12] emphasized that the environmental management activities of enterprises require the extensive participation of all employees, and without the support and cooperation of employees, the effect of environmental management is bound to be greatly reduced. Obviously, as most employees work in operational positions, they are well placed to identify and eliminate the processes that generate waste and harmful effluents [13], to identify and correct the incongruities in the production processes and green innovative designs, and to propose insightful green innovative solutions [4]. Therefore, in theory, employee incentives should be able to effectively stimulate employees’ environmental awareness and thus enhance the corporate environmental performance. Although Dahlmann et al. (2017) [8] found that including more beneficiaries from different levels within the corporate hierarchy is generally more likely to result in a reduction in corporate greenhouse gas emissions, we still know little about the micro-mechanisms behind the actual effects of employee incentives on corporate environmental performance. In an attempt to fill this gap, this paper intends to take advantage of the opportunity provided by the China Securities Regulatory Commission in restarting employee stock ownership plans (ESOPs) in 2014 to examine the impact of employee incentives on corporate environmental performance.

We chose China as our research context for two reasons. First, the rapid economic growth since China’s reforms and opening up has brought serious environmental pollution problems. As the 2018 Global Environmental Performance Index Report indicates, China’s environmental performance index ranked 120th out of 180 participating countries (regions), and air quality ranked fourth from the bottom. Severe air pollution in China is now responsible for 20% of deaths, killing 4000 people per day [1]. In response to environmental challenges, the Chinese government has implemented various environmental regulatory policies to reduce the environmental pollution caused by industrial production, and high-polluting enterprises are facing tremendous pressure to improve their environmental performance [14]. Our findings can, therefore, provide timely policy implications for enterprises in emerging economies facing similar problems. Second, in the US and many other developed countries, in addition to improving employee incentives, the implementation of ESOPs usually involves a variety of complex motives. For instance, ESOPs can be used by cash-constrained enterprises as a substitute for cash wages [15,16]. Employee stock options are tax deductible and hence are able to generate substantial non-debt tax shields [17,18]. Enterprises also use ESOPs to strengthen worker–management alliances to counter hostile takeovers [19,20]. However, ESOPs in China were mainly introduced as employee incentive programs [21,22]. China’s ESOPs are not tax deductible and they are not mixed with or altered by the effects of tax legislation or ESOPs pension programs [21]. Therefore, using China’s ESOPs to examine the impact of employee incentives on corporate environmental performance can obtain a cleaner conclusion.

Using Chinese high-polluting listed enterprises from 2014 to 2020, we provide evidence that ESOPs significantly improve corporate environmental performance and show that this finding is robust after a series of sensitivity tests. Moreover, we find that the positive effect of ESOPs on environmental performance is more pronounced in enterprises with weak free-riding problems, in enterprises with high human capital quality, and in non-state-owned enterprises. Our channel tests show that ESOPs improve corporate environmental performance through enhancing productivity and green technology.

This study makes several contributions to the extant literature. First, while previous studies on managerial incentives mainly emphasize the role of executive incentives in corporate environmental performance [5,6,7], our work focuses on ordinary employees who have become increasingly important in corporate environmental management activities. To the best of our knowledge, we are among the first to show how employee incentives influence corporate environmental performance using a large-scale analysis. Our results helps to make up for the shortcomings of existing studies and enrich the literature on managerial incentives. Second, we contribute to the literature on the economic consequences of ESOPs. The extant literature on the economic consequences of ESOPs mainly focuses on corporate performance [23], corporate governance [24], corporate innovation [18], equity return [25], and so on. Few studies pay attention to the impact of ESOPs on corporate environmental performance, especially the green effect of China’s ESOPs. Our results shed light on the micro-mechanisms behind the actual effects of ESOPs on corporate environmental performance, thereby enriching the literature on the economic consequences of ESOPs. In addition, our results show that the green effect of ESOPs depends on some specific situations. This provides timely implications for high-polluting enterprises in emerging markets concerned about the effects of corporate activities on the environmental performance and calls on enterprises to pay close attention to the impact of enterprise heterogeneity while implementing ESOPs to strengthen employee incentives.

The remainder of the paper proceeds as follows. Section 2 reviews the prior literature and develops our main hypothesis. Section 3 explains the data and variables. We analyze our main empirical results in Section 4 and report additional results in Section 5. Section 6 concludes the paper.

## 2. Institutional Background, Literature Review and Hypothesis Development

### 2.1. Institutional Background

The use of employee stock ownership plans in China first began with the joint-stock reform of state-owned enterprises in the 1990s. During this period, with the approval of the China Securities Regulatory Commission, state-owned enterprises could issue some equity to internal employees to improve operational efficiency. However, due to imperfect implementation details and a lack of effective supervision, the ESOPs not only failed to improve corporate performance [26], but also caused serious problems such as the transmission of benefits and the loss of state-owned assets [22]. For these reasons, the China Securities Regulatory Commission terminated the internal employee stock ownership of listed enterprises in December 1998. Since then, the shares held by internal employees of listed enterprises in China have gradually been converted into tradable shares.

On 20 June 2014, the China Securities Regulatory Commission issued the “Guiding Opinions on the Pilot Implementation of Employee Stock Ownership Plans by Listed Enterprises” (hereinafter referred to as the “Guiding Opinions”), which means that Chinese listed enterprises officially restarted the ESOPs. Additionally, the enterprises implementing the ESOPs are no longer limited to state-owned enterprises; private enterprises can also implement ESOPs according to their own needs without the special approval of the China Securities Regulatory Commission. Once the Guiding Opinions were issued, they immediately received a positive response from Chinese listed enterprises, and the number of state-owned listed enterprises and private listed enterprises that implemented ESOPs increased significantly. Different from the complex motivations for implementing ESOPs in the United States and other countries [20], China’ s ESOPs are a benefit-sharing mechanism in which employees enjoy the right to claim surplus by holding corporate stocks and a participation mechanism in which they have the right to make business decisions. It mainly emphasizes employee incentives, aiming to improve the benefit-sharing mechanism between workers and owners, improve employee cohesion and corporate competitiveness, and achieve optimal allocation of social capital. In terms of characteristics, although ESOPs and equity incentives are both long-term incentive system arrangements for enterprises, there are great differences in system design. First, in terms of implementation, the equity incentive plan is implemented for executives, and the scope is narrow; ESOPs for all employees cover a wide range. Second, the exercise conditions are different. The equity incentive plan takes the performance target as the exercise condition, and the employee stock ownership has no performance condition. Third, the management mode is different. In the equity incentive plan, executives can independently decide whether to unlock the stock or exercise their rights as long as the performance reaches the standard. The shares held by employees are uniformly managed by a management committee, and stock transactions need to be approved by vote of a shareholding committee. Therefore, it is difficult for employees to speculate in the short term, and more attention must be paid to the long-term value of the enterprise.

### 2.2. Literature Review

#### 2.2.1. Managerial Incentives and Environmental Performance

An upper-echelon perspective indicates that top managers have the decision-making power to influence the allocation of corporate resources; their attitudes and commitments play a crucial role in the selection and implementation of strategic decisions that ultimately affect environmental sustainability and performance [27]. Therefore, extant studies on managerial incentives mainly emphasize the role of executive incentives in sustainable development, examine how to restrain executives’ short-termism and hedonism, thereby stimulating executives’ willingness to take risks, and ultimately enhance corporate environmental governance practices and environmental performance [5,6,7]. For instance, Kock et al. (2012) [6] identified that enterprises offering higher market-based compensation to their CEOs show a superior level of environmental performance. Zou et al. (2015) [28] also found environmental incentives in executive compensation as having some positive impact on subsequent environmental performance. Kanashiro (2020) [29] argued that environmental compensation is a compelling incentive to motivate managers to invest in long-term and highly uncertain environmental strategies, which helps to lower toxic emissions by U.S. high-polluting enterprises. However, a survey conducted by Katsikeas et al. (2016) [30] with UK executives did not find an obvious impact of environmental performance incentives on eco-friendly product development strategies. Although many scholars have realized the significance of employees in corporate environmental management [9,10,12], Dahlmann et al. (2017) [8] also found that incentives that include multiple levels of beneficiaries, such as employees and executives, are more likely to reduce corporate greenhouse gas emissions. However, these studies do not consider the role of employee stock options in corporate environmental management activities; we still know little about the micro-mechanisms behind the actual effects of employee incentives on corporate environmental performance.

#### 2.2.2. A Review of ESOPs Studies

The research on ESOPs mainly focuses on two aspects. The first strand of literature seeks to reveal various motives for enterprises to implement ESOPs. For example, in addition to motivating employees, ESOPs can be used by cash-constrained enterprises to conserve cash flow [15,16,20]. Employee stock options are tax deductible and hence are able to generate substantial non-debt tax shields [17,18]. Enterprises may adopt ESOPs as an antitakeover device and increase wages to garner worker support [19,20]. Moreover, enterprises also use ESOPs to sort and retain certain types of employees [31]. The second strand of literature focuses on the economic functions of ESOPs. A handful of studies suggest that since the free-riding effect outweighs the incentive effect, ESOPs are just “incentives that have no incentive effects” [32]. However, most studies affirm the positive effects of ESOPs in terms of corporate governance [24], corporate performance [23], corporate innovation [18], and equity return [25]. For example, Jones and Kato (1995) [33] used panel data to estimate the production function and reported that the introduction of employee ownership has led to an average 4–5% increase in Japanese corporate productivity. Chang et al. (2015) [18] provided empirical evidence that non-executive employee stock options have a positive impact on corporate innovation. Zhou et al. (2022) [22] found that ESOPs improve corporate CSR performance by providing employees with external economic incentives and internal psychological incentives.

Notably, although the current research results in related fields have been abundant, there are still some limitations worthy of further study. First, existing studies on the role that managerial incentives play in improving corporate environmental performance have so far focused on incentives provided either to executives and senior managers or to plant managers, yet few studies have considered the role of employee incentives. Second, few studies have explored the role of ESOPs in corporate sustainable development, especially in emerging market economies such as China. Drawing on the opportunity provided by the China Securities Regulatory Commission in restarting the ESOPs in 2014, this paper investigates the impact of ESOPs on the environmental performance of high-polluting enterprises, which not only helps to make up for the shortcomings of the existing literature in theory, but also provides important policy implications for enterprises to timely enhance their environmental performance in practice.

### 2.3. Hypothesis Development

Productivity and green technology are two important factors affecting corporate environmental performance. The higher the productivity, the less energy resources and toxic emissions per unit of output, and the better the corporate environmental performance [34,35,36]. For example, Bloom et al. (2010) [34] and Cui et al. (2015) [37], using data from British and American manufacturing enterprises, respectively, found that corporate productivity significantly reduces the intensity of pollution emissions per unit of output. Barrows and Ollivier (2018) [36] used theoretical models to clearly show that there is a negative correlation between corporate productivity and pollution emission intensity, and confirmed this conclusion with pollution emission data on Indian enterprises. Green technology represents the efficiency of energy resource utilization and the pollution control ability of enterprises. A higher level of green technology means that enterprises can conserve the energy resources they consume, lower their production of toxic substances and reduce the emission of pollutants in the production process [14,38]. For example, using data from Chinese listed enterprises, Long et al. (2022) [2] and Ma et al. (2022) [38] found that green technology is an important driver for improving corporate environmental performance. Based on previous studies, we hold that ESOPs improve corporate environmental performance through enhancing productivity and green technology. This is mainly due to the following reasons.

First, before the implementation of the ESOPs, it was difficult for employees to enjoy the benefits brought by improvements in corporate environmental performance, such as the direct economic benefits brought by rising stock prices and the indirect economic benefits brought by improved corporate green reputations [22]. On the contrary, they had to bear more risks caused by failures of environmental strategy, such as layoffs due to a decline in performance. As a result, the mismatch of risk–returns may have resulted in employees slacking off, making only limited effort equivalent to their fixed compensation rather than exerting extra effort [20]. After the implementation of the ESOPs, employees were transformed from migrant workers receiving fixed salaries to owners enjoying corporate ownership and surplus income sharing rights, which directly enhanced employees’ satisfaction and dedication, stimulated their sense of ownership [22], and encouraged them to work harder and actively participate in corporate environmental management activities, such as actively identifying and eliminating processes that generate waste and harmful effluents [13], identifying and correcting incongruities in production processes and green innovative designs, and proposing insightful green innovative solutions [4]. Increased efforts by employees can help enhance the productivity and green innovation efficiency of enterprises [18,21], thus improving corporate environmental performance.

Second, unlike conventional corporate investment projects, green technology innovation projects are long-term, multi-stage, and labor-intensive [39]. Moreover, they involve a high risk of failure due to their dependence on various unpredictable conditions [29]. Therefore, green technology innovation requires continuous and stable human capital investment, and the departure of core employees usually decreases the probability of success of corporate innovation projects. Employee stock options normally have a long vesting period and a long average time to expiration. To sufficiently benefit from ESOPs, employees have to stay with their enterprises for many years [16]. This feature of ESOPs further enhances the loyalty of employees, reduces employee turnover, encourages employees’ long-term human capital investment in innovation [18,21], and ultimately improves corporate environmental performance by enhancing productivity and green technology.

Finally, ESOPs bind the enterprises’ interests more closely to employees’ wealth, thus inducing mutual monitoring between employees and enhancing cooperation among co-workers. On the one hand, ESOPs make each employee’s actions affect payments to other members of the group, as lazy and careless behavior by individual employees can lead to damage to collective interests; hence, other employees have a strong incentive to monitor and sanction such irresponsible behavior [23]. For example, Freeman et al. (2010) [40] surveyed over 40,000 employees from 14 enterprises with ESOPs. The survey results showed that those with corporate stock were much more likely to choose to “talk directly to the employee” or “speak to a supervisor” rather than “do nothing”. On the other hand, the collective incentive to expand the economic cake helps to stimulate and support employees’ knowledge-sharing routines, information absorption and skill transmission regarding environmental protection [8,30]. Most importantly, existing research has shown that mutual supervision and cooperative learning among employees can help enhance the productivity and green technology of enterprises [18,20,21], thereby improving corporate environmental performance.

Based on the above analysis, we propose the main hypothesis of this paper, as follows:

**Hypothesis** **1:** *ESOPs help improve corporate environmental performance.*

## 3. Data and Methodology

### 3.1. Data and Sample

To investigate the relationship between ESOPs and corporate environmental performance, we constructed our sample based on Chinese listed enterprises in high-polluting industries from 2014 to 2020 (We follow the categories of the “Guide to Environmental Information Disclosure of Listed Enterprises” issued by the Ministry of Environmental Protection of the People’s Republic of China in 2010. The high-polluting industries involve mining, textiles, paper making and paper products, petroleum, chemical, chemical fiber, black (non-ferrous) metal smelting and processing, rubber and plastic, pharmaceutical, fur products and other industries). We chose high-polluting enterprises as the research object because the activities of high-polluting enterprises are the primary source of pollution [1]. Our sample period began in 2014 because the China Securities Regulatory Commission restarted the ESOPs in 2014. Our data were obtained from three sources: (1) ESOPs data were obtained from the China Research Data Service Platform (CNRDS) database; (2) corporate environmental performance data were collected from annual financial reports; (3) corporate financial data and other data were obtained from the China Stock Market and Accounting Research database (CSMAR). Following prior studies [2,22], we performed the following procedure to filter the data: winsorizing all continuous variables; deleting all outliers, such that the total number of assets is negative, and asset–liability ratio is higher than 1; excluding cross-listed enterprises; eliminating Special Treatment enterprises, and dropping enterprises with missing data. In addition, we excluded enterprises with discontinued ESOPs. Finally, we obtained 4607 enterprise-year observations for 798 listed high-polluting enterprises, among which 234 enterprises implemented ESOPs during the sample period. Appendix A presents the distribution of the number of companies and employees by sector, ownership, etc. in 2014 and 2020.

### 3.2. Methodology and Variables

To examine the relationship between ESOPs and corporate environmental performance, following Kim and Ouimet (2014) [20] and Zhou et al. (2022) [22], we formulated the following basic regression model:

(1) $$PP=\beta_{0}+\beta_{1}ESOP+\beta_{2}X+\lambda +\mu +\varepsilon$$

where the dependent variable *PP* denotes the corporate environmental performance. There are many measures of *PP*, but no unified standard has been established. China‘s sewage charges involve all solid, liquid, gas and other harmful pollutants, mainly including sewage charges, waste gas charges, solid waste and hazardous waste charges and excessive noise charges. The sewage charges paid by enterprises are directly related to the types and quantities of various pollutants discharged. As the collection standard for sewage charges involves the type and quantity of all kinds of pollutants emitted by enterprises, which can comprehensively reflect their pollution emissions, this index can overcome the shortcomings of other indicators that are not comprehensive enough. The more sewage charges an enterprise is compelled to pay, the worse its *PP*. Therefore, following He et al. (2022) [41], we used sewage charges to measure *PP* and standardized sewage charges with operating income. To enhance the readability of the regression coefficient, the standardized sewage charges were multiplied by 1000.

The independent variable *ESOP* is a dummy variable that equals 1 if enterprises implement ESOPs in that year; otherwise 0. *X* is a vector of the control variables. Referring to Long et al. (2022) [2] and Wang et al. (2022) [14], all variable definitions are presented in Table 1, including *Size*, *Lev*, *Roa*, *Growth*, *Cash*, *PPE*, *Age*, *Soe*, *Mshare*, *Top1*, *Board*, and *Dual*. *λ* and *μ* are the fixed effects for year and firm. *ε* is an error term.

Table 1 shows the definition of variables in this study.

### 3.3. Descriptive Statistics and Correlations

Table 2 reports the descriptive statistics for the main variables of our basic regression model. The mean value, standard deviation, and maximum value of *PP* were 0.290, 1.035, and 7.453, respectively. The mean value of *ESOP* was 0.051, indicating that only approximately 5.1% of the enterprises in our sample had ESOPs. The mean value, standard deviation, and maximum value of *Size* were 22.295, 1.233, and 25.996, respectively; those of *Lev* were 0.394, 0.198, and 0.902, respectively; those of *Roa* were 0.041, 0.062, and 0.206, respectively; and those of *Growth* were 0.134, 0.357, and 2.140, respectively. These findings indicate that the values of *Size*, *Lev*, *Roa*, and *Growth* were quite different among the listed high-polluting enterprises in our sample. In addition, these results are similar to the findings of previous studies [1,2].

Table 3 reports the correlation coefficients of the main variables. As revealed in the table, the absolute values of the correlation coefficients between all variables were less than 0.5. This indicates that there was no serious multicollinearity between the variables. Moreover, *ESOP* and *PP* were significantly negatively correlated, preliminarily indicating that ESOPs improve corporate environmental performance.

## 4. Empirical Results and Analysis

### 4.1. Baseline Regression

Table 4 reports the results of our baseline regressions in Equation (1). Column (1) estimates the basic impact of ESOPs on corporate environmental performance while controlling for fixed effects of year and firm. The coefficient of *ESOP* was −0.102 and significantly negative, indicating that ESOPs improve corporate environmental performance. Column (2) reports regressions that control for various variables as well as year and firm fixed effects. We found that the coefficient of *ESOP* was −0.099 and significantly negative at the 5% level, consistent with the above conclusion. All of our results showed that ESOPs can improve corporate environmental performance, and Hypothesis 1 is supported by the empirical results.

### 4.2. Endogeneity

Although we documented a strongly positive association between ESOPs and corporate environmental performance, the results were potentially subject to two types of endogeneity. The first type is omitted variable bias. While we controlled for a standard set of variables that have been shown by previous studies to affect corporate environmental performance, the relation that we observed may be spurious if our model omitted any variables that affect both ESOPs and corporate environmental performance. For example, enterprises with high management quality are more likely to introduce ESOPs. At the same time, enterprises with high management quality may pay more attention to environmental management activities, and the corresponding environmental performance will be better. The second possible endogeneity issue is reverse causality running from corporate environmental performance to ESOPs. The causal relationship between ownership structure and enterprise characteristics is difficult to identify [42], and research on the relationship between ownership structure and environmental performance is susceptible to reverse causality. Determining whether ESOPs improve environmental performance or whether enterprises with better environmental performance are more concerned with the employee’s interests and, therefore, implement ESOPs is also a difficult task. Hence, following previous studies [1,18,22], we used the propensity score matching and difference-in-differences model (PSM + DID) and added potentially omitted variables to mitigate the above endogenous concerns, respectively.

#### 4.2.1. Propensity Score Matching and Difference-in-Differences Model (PSM + DID)

As an exogenous event, the restart of employee stock ownership plans in 2014 by the China Securities Regulatory Commission provides a good quasi-natural experimental scenario for this paper to examine differences in environmental performance between the experimental group and the control group before and after the event. To this end, we extended the sample period from 2010 to 2020 to facilitate the difference-in-differences test. First, we defined the enterprises that implemented ESOPs during the sample period as the experimental group, and the enterprises that did not implement ESOPs as the control group. We used the propensity score matching (PSM) method to alleviate systematic differences between the experimental group and control group. Specifically, this paper used the neighboring matching method (1:2) to match the most appropriate control group samples with the experimental group samples, and all control variables in the baseline regression were used as the matching criteria. Appendix B tabulates the results of equilibrium hypothesis testing in detail. It can be seen that there was a huge difference between the experimental group and control group before matching. After matching, the difference was no longer significant, which indicates the selection of matching variables and matching methods was reasonable.

Second, we constructed the following DID model to test the impact of ESOPs on corporate environmental performance:

(2) $$PP=\beta_{0}+\beta_{1}Treat\;*\;Post+\beta_{2}Treat+\beta_{3}X+\lambda +\mu +\varepsilon$$

where *Treat* is the dummy variable of the experimental group; *Post* is the time dummy variable, which equals 1 after enterprises implement ESOPs. The interaction of *Treat* and *Post* (*Treat*∗*Post*) measures the absolute effect of ESOPs on corporate environmental performance. Other variables are defined the same as those in Equation (1). Column (1) in Table 5 reports the estimates of Equation (2). The coefficient of *Treat*∗*Post* was significantly negative, suggesting that ESOPs help improve corporate environmental performance. Moreover, the results also ruled out the impact of reverse causality problems to some extent.

#### 4.2.2. Adding Potentially Omitted Variables

First, prior studies found that environmental regulation can improve corporate environmental performance and affect the equity incentive mechanism of enterprises [43,44]. Thus, referring to Li et al. (2020) [44], we used the proportion of regional pollution control investment in regional GDP to measure environmental regulation (*ER*) and added *ER* as a control variable to Equation (1). Second, because equity incentive plans may also influence corporate sustainability practices and environmental performance [45], we further controlled the dummy variable of whether the corporate implements equity incentive plans (*EIPs*) in Equation (1). Third, management quality may affect both ESOPs and corporate environmental performance [46,47]. Referring to Zhou and Kim (2021) [48], we used the DIB internal control index (*DIBindex*) to measure the quality of management and added *DIBindex* as a control variable to Equation (1). Finally, following Zhang et al. (2019) [1], we also controlled for region-by-year fixed effects and industry-by-year fixed effects to remove any time-variant shocks at regional and industry levels, respectively. Column (2) in Table 5 reports the estimates of adding the above control variables. The coefficient of *ESOP* is significantly negative at the 10% level, consistent with the baseline regression results.

### 4.3. Other Robustness Tests

We ran a variety of other robustness tests to ensure the validity of our findings.

First, to examine the sensitivity of our results for a specific measure, we conducted robustness checks by introducing an alternative dependent variable. Following Zhang et al. (2019) [1], we used corporate environmental investment (*CEI*) to measure corporate environmental performance, and we eliminated the scale effect by using the operating income to standardize environmental investment. Column (3) in Table 5 reports the results of the regression analysis. The estimated coefficient of *ESOP* was significantly positive, indicating that ESOPs enhance environmental investment by enterprises. Therefore, Hypothesis 1 is still supported. Second, the results may be affected by the measure of ESOPs. Thus, we further used the ratio of employee shareholding to total shares (*ESPO_ZB*) to measure ESOPs. Column (4) in Table 5 reports the regression results. The estimated coefficient of *ESPO_ZB* was significantly negative, indicating that the conclusion is still valid. Third, we adjusted the sample. On the one hand, to alleviate the interference of equity incentives in our findings, following Zhou et al. (2022) [22], we excluded the enterprises with equity incentive plans. On the other hand, we further expanded the sample from high-polluting enterprises to all A-share listed enterprises. Columns (5) and (6) in Table 5 report the regression results, respectively. The estimated coefficients of *ESOP* were significantly negative, which proves once again that our conclusion is robust.

## 5. Further Analysis

### 5.1. Cross-Sectional Tests

In this subsection, we partitioned our sample in several ways to investigate whether the effect of ESOPs on corporate environmental performance varied across enterprises.

#### 5.1.1. Free-Riding among Employees

The free-riding problem holds that when there are many employees, individual workers may feel they have little impact on the overall output and hence not exert additional efforts, thus damaging the economic cake of the enterprise [20]. This free-rider effect, often referred to as the 1/*N* effect, intensifies as the number of employees, *N*, increases. Many of the relevant studies also confirm that the free-riding problem can weaken the incentive effect of ESOPs and reduce the positive impact of employee incentives on corporate productivity and innovation levels [18,23]. While we have found that ESOPs form a strong employee incentive device to improve environmental performance, the power of this employee incentive can be diluted if free-riding problems are severe among employees. Hence, we expect that the positive impact of ESOPs on environmental performance is more pronounced for enterprises with a weak free-riding problem. In view of the fact that the overall enterprise success with fewer employees is more sensitive to the actions of individual workers [18], the wealth of employees is also more closely related to the interests of the enterprise. Moreover, the enterprise with fewer employees usually has more effective control systems; the overall corporate supervision mechanism and the mutual supervision between employees can detect and prevent the lazy and careless behavior of individual employees in time, thereby reducing the free-riding problem [20]. Therefore, using the number of employees to measure the free-riding problem has become a common method in the literature. Following previous studies [18,20,23], we separated enterprises into subsamples using the number of employees as a proxy for the extent of free-riding among employees. Enterprises with above (below) the median number of employees were defined as having severe (weak) free-riding problems. Columns (1) and (2) in Table 6 report the regression results. Obviously, the effect of ESOPs on environmental performance is indeed more pronounced among enterprises with weak free-riding problems.

#### 5.1.2. Human Capital Quality

The impact of ESOPs on corporate environmental performance depends not only on employees’ efforts, but also on employees’ knowledge reserves and skill levels. When the quality of human capital is higher, the marginal effect of increased employee efforts is greater, and the impact on corporate environmental performance will be more intuitive. So, we expect that employee incentives provided by ESOPs have a stronger impact on the environmental performance in enterprises where the quality of human capital is relatively high. Referring to Escribano et al. (2009) [49], we used the proportion of technical employees among total employees to measure human capital quality, and we classified enterprises with a proportion of technical employees among total employees above (below) the sample median as having high (low) human capital quality. We then recalculated with Equation (1) for the two groups separately. Columns (3) and (4) in Table 6 report the regression results. The results show that the effect of ESOPs on environmental performance is more pronounced in enterprises with high human capital quality, which is in line with the above expectation.

#### 5.1.3. Ownership Structure

Differences in enterprise ownership are a prominent feature of China’s economic system. We further tested whether ownership structure affects the relationship between ESOPs and corporate environmental performance. On the one hand, compared with non-state-owned enterprises (non-SOEs), state-owned enterprises (SOEs) usually bear more social responsibilities that compel them to pay more attention to environmental protection [14,50]. As a result, ESOPs in SOEs are more likely to motivate employees to make extra efforts in environmental protection, and the improvement in corporate environmental performance is more obvious. On the other hand, the implementation of ESOPs by SOEs faces the double restriction of employee scope and shareholding ratio, which makes the incentive effect of ESOPs in SOEs significantly weaker than that in non-SOEs. Moreover, SOEs have lower management quality, and the link between their ESOPs and environmental performance may be weaker. In addition, the treatment and welfare of employees in SOEs are relatively better, so the incentive effect of ESOPs will be further weakened. Therefore, the effect of ESOPs on corporate environmental performance may also be more significant in non-SOEs. Columns (5) and (6) in Table 6 present the regression results for the effect of ESOPs on the corporate environmental performance of SOEs and non-SOEs, respectively. We found that the coefficient of *ESOP* was negative and significant in the subsample of non-SOEs, yet insignificant in the subsample of SOEs, indicating that the effect of ESOPs on environmental performance is more pronounced in non-SOEs.

### 5.2. Mechanism Analysis

Thus far, we have confirmed that ESOPs significantly improve corporate environmental performance. In this subsection, we turn to investigate the underlying mechanisms behind our conclusions. According to the previous analysis, productivity and green technology are two important factors affecting corporate environmental performance, and ESOPs can improve corporate environmental performance through enhancing productivity and green technology. To provide further evidence of support, we conducted an empirical test of these two channels.

First, following Wang et al. (2022) [14], we used the total factor productivity (*TFP*) estimated by the LP method to measure the productivity of enterprises. Then, we replaced the dependent variable of the baseline regressions with *TFP*. Column (1) in Table 7 reports the results of the regression analysis. The estimated coefficient of *ESOP* was 0.055 and significantly positive at the 1% level, thus providing empirical evidence that ESOPs improve corporate environmental performance by enhancing productivity. Second, following Long et al. (2022) [2] and Wang et al. (2022) [14], we used green patent applications (*GP*) as a proxy for the level of corporate green technology, and *GP* wa measured as the natural logarithm of total green patent applications plus 1. We then replaced the dependent variable of the baseline regressions with *GP*. Column (2) in Table 7 reports the results of the regression analysis. The estimated coefficient of *ESOP* was 0.016 and significantly positive at the 5% level, thus providing empirical evidence that ESOPs improve corporate environmental performance by enhancing green technology. To sum up, these findings indicate that ESOPs improve corporate environmental performance through enhancing productivity and green technology and also reveal the micro-mechanisms behind the actual effects of employee incentives on corporate environmental management.

## 6. Discussion, Conclusions and Policy Implications

### 6.1. Discussion

Although the importance of employees in corporate environmental management activities has been affirmed by a large amount of literature [4,9,10,12], the extant literature on managerial incentives mainly focuses on executive incentives, and few works in the literature examine the role of employee incentives in corporate environmental performance. Drawing on the opportunity afforded by the China Securities Regulatory Commission in restarting employee stock ownership plans (ESOPs) in 2014, this paper investigated the impact of employee incentives on the environmental performance of high-polluting enterprises and its micro-mechanisms. This paper makes two contributions to the extant literature. First, to the best of our knowledge, we are among the first to show how employee incentives influence corporate environmental performance using a large-scale analysis. Our results help to make up for the shortcomings of existing studies and enrich the literature on managerial incentives. Second, few studies pay attention to the impact of ESOPs on corporate environmental performance, especially the green effect of ESOPs in China. Our results shed light on the micro-mechanisms behind the actual effects of ESOPs on corporate environmental performance, thereby enriching the literature on the economic consequences of ESOPs. In addition, this study also provides timely policy recommendations for high-polluting enterprises in emerging markets such as China to effectively improve environmental performance, that is, enterprises should pay attention to enterprise heterogeneity while attaching importance to employee incentives, and try to avoid the negative impacts of the free-riding problem, enterprise ownership and low human capital quality.

Although this study provides some new insights, there are still some limitations to be further studied in the future. First, this paper mainly interpreted how employee incentives affect corporate environmental performance from the perspective of productivity and green technology. Although this has certain support in the literature, it may still ignore other possible impact mechanisms. In the future, we can consider examining the impact of employee incentives on corporate environmental management activities from other perspectives. Second, the indicators for measuring corporate environmental performance have not yet gained a consensus. Although this paper referred to He et al. (2022) [41] and Zhang et al. (2019) [1], using sewage charges and corporate environmental investment to measure corporate environmental performance, it is not clear whether this measurement method is applicable to developed countries and other emerging market economies. Therefore, future research may consider using more measurement methods to verify the positive role of employee incentives in enterprise environmental management. Third, the research sample in this paper was limited to China, and employee stock ownership plans were used to characterize employee incentives, which may reduce the universality of the conclusions of this paper to some extent. Therefore, future research can adopt cross-country data and use other employee incentive systems to examine the impact of ordinary employee incentives on corporate environmental performance. Finally, we mainly used the data on high-polluting enterprises to examine the impact of ESOPs on corporate environmental performance, but it is worth noting that in our sample, the proportion of state-owned enterprises was about 34%, and the proportion of enterprises in the chemical and pharmaceutical sectors was about 56%, which means our conclusion may have been affected by the sample distribution. Therefore, future research can consider using more balanced samples or cleaner scenarios to investigate the relationship between employee incentives and corporate environmental performance.

### 6.2. Conclusions

Drawing on the opportunity provided by the China Securities Regulatory Commission in restarting employee stock ownership plans (ESOPs) in 2014, this paper examined the impact of ESOPs on the environmental performance of high-polluting enterprises using data on Chinese high-polluting listed enterprises from 2014 to 2020. The main conclusions are as follows: (1) ESOPs can help improve the environmental performance of high-polluting enterprises. (2) This effect is stronger for enterprises with weak free-riding problems, for non-state-owned enterprises, and for enterprises that have high human capital quality. (3) Mechanism tests show that ESOPs improve corporate environmental performance by enhancing productivity and green technology.

### 6.3. Policy Implications

Our findings have important implications for high-polluting enterprises and policy makers. First, as stakeholders such as the public, investors, customers, suppliers and governments pay more attention to environmental pollution, the findings of our studies may be useful for enterprises in planning and promoting environmental performance to prevent loss of legitimacy and stakeholder support. These results suggest that ESOPs can be used by high-polluting enterprises in China as an effective strategic tool to enhance productivity and green technology, and then actively improve corporate environmental performance. In addition, we found that the positive effect of ESOPs on environmental performance varies significantly across enterprises. Therefore, enterprises should fully consider their own actual situation when designing and implementing employee incentive systems to avoid the negative impact of free-riding, low quality of human capital, and state-owned equity. Second, our research provides valuable insights and suggestions for the government. Government departments in emerging market economies such as China should further implement and improve the employee incentive system represented by ESOPs and fully develop and mobilize the enthusiasm of employees in corporate environmental protection strategies, so as to achieve economic and environmental goals simultaneously.

## Figures and Tables

**Table 1:** Variable definitions.

Variable Type	Variable Name	Variable Definition
Dependent variable	*PP*	Sewage charges divided by total assets, multiplied by 1000
Independent variable	*ESOP*	Dummy variable, equals 1 if enterprise implements ESOPs in that year; otherwise 0
Control variable	*Size*	The natural logarithm of total assets
	*Lev*	Total liabilities divided by total assets
	*Roa*	Net profit divided by total assets
	*Growth*	The growth rate of net profit.
	*Cash*	Cash equivalent divided by total assets
	*PPE*	Fixed assets divided by total assets
	*Age*	The natural logarithm of the year of the firm’s establishment
	*Soe*	A variable that equals 1 if the enterprise is a state-owned enterprise; otherwise 0
	*Mshare*	The proportion of management shareholding
	*Top1*	The proportion of the largest shareholder
	*Board*	The natural logarithm of the number of board directors.
	*Dual*	Dummy variable; duality indicates the combination of the chairman of the board and chief executive officer (CEO); otherwise, it is 0.

**Table 2:** Descriptive statistics.

Variable	Observations	Mean	S.D.	Minimum	Median	Maximum
*PP*	4607	0.290	1.035	0.000	0.000	7.453
*ESOP*	4607	0.051	0.220	0.000	0.000	1.000
*Size*	4607	22.295	1.233	20.142	22.115	25.996
*Lev*	4607	0.394	0.198	0.055	0.380	0.902
*Roa*	4607	0.041	0.062	−0.212	0.039	0.206
*Growth*	4607	0.134	0.357	−0.514	0.081	2.140
*Cash*	4607	0.132	0.100	0.009	0.104	0.493
*Tangibility*	4607	0.279	0.145	0.030	0.262	0.664
*Age*	4607	2.263	0.720	0.693	2.398	3.258
*Soe*	4607	0.340	0.474	0.000	0.000	1.000
*Mshare*	4607	0.748	0.434	0.000	1.000	1.000
*Top1*	4607	34.145	14.291	9.670	31.940	74.980
*Board*	4607	2.218	0.240	1.609	2.197	2.890
*Dual*	4607	0.265	0.441	0.000	0.000	1.000

Note: Table 2 presents descriptive statistics of main variables. All of the variables are defined in Table 1.

**Table 3:** Correlation coefficient.

Variables	*PP*	*ESOP*	*Size*	*Lev*	*Roa*	*Growth*	*Cash*	*Tangibility*	*Age*	*Soe*	*Mshare*	*Top1*	*Board*	*Dual*
*PP*	1	−0.027 *	0.131 ***	0.135 ***	−0.088 ***	−0.038 ***	−0.168 ***	0.271 ***	0.114 ***	0.107 ***	−0.049 ***	0.043 ***	0.037 **	−0.063 ***
*ESOP*	−0.026 *	1	0.030 **	0.002	0.041 ***	0.056 ***	−0.000	−0.031 **	−0.048 ***	−0.099 ***	0.034 **	0.007	−0.050 ***	0.029 **
*Size*	−0.010	0.023	1	0.478 ***	−0.118 ***	0.003	−0.241 ***	0.227 ***	0.411 ***	0.346 ***	−0.069 ***	0.216 ***	0.232 ***	−0.182 ***
*Lev*	0.072 ***	−0.001	0.474 ***	1	−0.470* **	−0.061 ***	−0.412 ***	0.305***	0.308 ***	0.291***	−0.115 ***	0.051 ***	0.149 ***	−0.107 ***
*Roa*	−0.035 **	0.028 *	−0.061 ***	−0.432 ***	1	0.361 ***	0.338 ***	−0.217***	−0.238 ***	−0.242***	0.140 ***	0.071 ***	−0.075 ***	0.117 ***
*Growth*	−0.016	0.035 **	0.030 **	−0.032 **	0.281 ***	1	0.055 ***	−0.083***	−0.179 ***	−0.129***	0.078 ***	−0.024 *	−0.040 ***	0.064 ***
*Cash*	−0.080 ***	−0.009	−0.238 ***	−0.406 ***	0.294 ***	0.022	1	−0.376***	−0.133 ***	−0.057***	0.019	0.031 **	−0.023	0.042 ***
*Tangibility*	0.162 ***	−0.03 5**	0.264 ***	0.321 ***	−0.187 ***	−0.059 ***	−0.371 ***	1	0.159 ***	0.279***	−0.100 ***	0.130 ***	0.118 ***	−0.118 ***
*Age*	0.022	−0.037 **	0.415 ***	0.328 ***	−0.199 ***	−0.110 ***	−0.154 ***	0.188***	1	0.512***	−0.287 ***	−0.004	0.171 ***	−0.231 ***
*Soe*	0.053 ***	−0.099 ***	0.380 ***	0.308 ***	−0.168 ***	−0.092 ***	−0.066 ***	0.297***	0.493 ***	1	−0.336 ***	0.249 ***	0.263 ***	−0.260 ***
*Mshare*	−0.016	0.034 **	−0.082 ***	−0.131 ***	0.105 ***	0.017	0.009	−0.116***	−0.271 ***	−0.336***	1	−0.155 ***	−0.055 ***	0.161 ***
*Top1*	−0.010	0.004	0.289 ***	0.063 ***	0.088* **	0.007	0.024 *	0.139***	−0.013	0.261***	−0.171 ***	1	0.048 ***	−0.020
*Board*	0.020	−0.040 ***	0.256 ***	0.166 ***	−0.056 ***	−0.009	−0.034 **	0.123***	0.171 ***	0.268***	−0.061 ***	0.061 ***	1	−0.157 ***
*Dual*	−0.004	0.029 **	−0.179 ***	−0.115 ***	0.091 ***	0.038 ***	0.038 ***	−0.121***	−0.239 ***	−0.260***	0.161 ***	−0.037 **	−0.151 ***	1

Note: Lower triangular cells report Pearson’s correlation coefficients, upper triangular cells are Spearman’s rank correlation. ***, **, and * represent statistical significance at the 1%, 5%, and 10% levels, respectively.

**Table 4:** Employee stock ownership plans and corporate environmental performance.

Variable	(1)	(2)
	*PP*	*PP*
*ESOP*	−0.102 **	−0.099 **
	(−2.261)	(−2.197)
*Size*		−0.100 *
		(−1.674)
*Lev*		0.417 **
		(2.185)
*Roa*		0.325
		(0.925)
*Growth*		−0.049
		(−1.467)
*Cash*		−0.147
		(−0.678)
*Tangibility*		0.189
		(0.805)
*Age*		−0.024
		(−0.241)
*Soe*		0.535 ***
		(4.247)
*Mshare*		−0.003
		(−0.058)
*Top1*		0.000
		(0.037)
*Board*		0.021
		(0.308)
*Dual*		0.012
		(0.245)
*Constant*	0.540 ***	2.323 *
	(14.135)	(1.844)
*Year Fixed Effects*	Yes	Yes
*Firm Fixed Effects*	Yes	Yes
*R-squared*	0.424	0.428
*Observations*	4607	4607

Note: t-statistics are reported in parentheses. ***, **, and * represent statistical significance at the 1%, 5%, and 10% levels, respectively.

**Table 5:** Robustness tests.

Variable	(1)	(2)	(3)	(4)	(5)	(6)
	*PP*	*PP*	*CEI*	*PP*	*PP*	*PP*
*ESOP*		−0.079 *	0.154 *		−0.120 ***	−0.018 *
		(−1.681)	(1.949)		(−2.799)	(−1.752)
*ESPO_ZB*				−0.071 **		
				(−2.523)		
Treat*Post	−0.015 **					
	(−2.112)					
*Treat*	0.007					
	(1.233)					
*Size*	0.003	−0.097 *	0.107 *	−0.102 *	−0.085	−0.017
	(1.310)	(−1.699)	(1.691)	(−1.712)	(−1.360)	(−1.528)
*Lev*	0.014	0.329 *	0.164	0.414 **	0.458 **	0.103 **
	(1.190)	(1.830)	(1.029)	(2.173)	(2.285)	(2.478)
*Roa*	−0.085 *	0.209	0.055	0.313	0.574	−0.233 ***
	(−1.865)	(0.601)	(0.197)	(0.891)	(1.514)	(−4.096)
*Growth*	−0.005	−0.049	−0.082 *	−0.048	−0.070 **	−0.018 ***
	(−1.299)	(−1.456)	(−1.915)	(−1.443)	(−2.015)	(−3.088)
*Cash*	0.035	0.039	0.285	−0.148	−0.156	−0.097 **
	(0.929)	(0.177)	(1.321)	(−0.678)	(−0.659)	(−2.314)
*Tangibility*	0.120 ***	0.168	0.659 **	0.185	0.118	0.195 ***
	(7.266)	(0.664)	(2.512)	(0.788)	(0.478)	(3.104)
*Age*	−0.005	0.070	0.064	−0.024	0.032	0.020
	(−1.408)	(0.541)	(0.540)	(−0.233)	(0.302)	(1.086)
*Soe*	−0.002	0.525 ***	0.135	0.532 ***	0.535 ***	0.077 ***
	(−0.357)	(4.108)	(1.134)	(4.222)	(3.943)	(2.975)
*Mshare*	−0.006	−0.006	−0.026	−0.003	−0.002	−0.008
	(−1.125)	(−0.146)	(−0.494)	(−0.077)	(−0.051)	(−0.689)
*Top1*	−0.000 **	−0.000	−0.003	0.000	−0.001	−0.000
	(−2.463)	(−0.108)	(−0.764)	(0.075)	(−0.388)	(−0.311)
*Board*	−0.010	0.046	−0.093	0.020	0.026	0.006
	(−1.252)	(0.656)	(−1.235)	(0.293)	(0.359)	(0.374)
*Dual*	0.006	0.031	−0.017	0.012	0.044	0.015
	(1.066)	(0.601)	(−0.355)	(0.236)	(0.820)	(1.537)
*ER*		−0.585				
		(−0.431)				
*EIPs*		0.140 ***				
		(2.734)				
*DIBindex*		0.002				
		(0.165)				
*Constant*	−0.011	2.121 *	−2.134 *	2.373 *	1.866	0.422 *
	(−0.276)	(1.719)	(−1.664)	(1.883)	(1.408)	(1.694)
*Year Fixed Effects*	Yes	Yes	Yes	Yes	Yes	Yes
*Firm Fixed Effects*	Yes	Yes	Yes	Yes	Yes	Yes
*Ind*∗*Year Fixed Effects*	No	Yes	No	No	No	No
*Region*∗*Year Fixed Effects*	No	Yes	No	No	No	No
*R-squared*	0.063	0.450	0.362	0.428	0.410	0.415
*Observations*	3525	4607	4607	4607	4123	19,149

Note: t-statistics are reported in parentheses. ***, **, and * represent statistical significance at the 1%, 5%, and 10% levels, respectively.

**Table 6:** Cross-sectional tests.

Variable	(1)	(2)	(3)	(4)	(5)	(6)
	PP	PP	PP	PP	PP	PP
	Free-Riding Problem		Human Capital Quality		Ownership Structure	
	High	Low	High	Low	SOEs	Non-SOEs
*ESOP*	−0.062	−0.115 **	−0.212 **	−0.047	−0.025	−0.101 **
	(−0.905)	(−2.304)	(−2.220)	(−0.931)	(−0.303)	(−2.027)
*Size*	0.102 **	−0.037	−0.044	−0.204 ***	−0.144	−0.084
	(2.411)	(−0.498)	(−0.317)	(−3.084)	(−1.121)	(−1.495)
*Lev*	−0.180	0.725 **	−0.053	0.828 ***	1.171 ***	0.191
	(−0.721)	(2.574)	(−0.138)	(2.980)	(2.600)	(1.008)
*Roa*	0.020	−0.130	0.908	0.252	1.142	−0.097
	(0.047)	(−0.266)	(1.373)	(0.529)	(1.481)	(−0.268)
*Growth*	0.001	−0.038	−0.092	−0.023	−0.089	−0.008
	(0.018)	(−0.859)	(−1.511)	(−0.521)	(−1.426)	(−0.220)
*Cash*	0.074	−0.043	0.214	0.039	−0.052	0.033
	(0.269)	(−0.159)	(0.382)	(0.148)	(−0.104)	(0.143)
*Tangibility*	0.095	0.024	0.485	0.573 *	0.179	0.381
	(0.334)	(0.072)	(1.004)	(1.733)	(0.453)	(1.266)
*Age*	−0.230	−0.002	−0.368 *	0.215 *	−0.058	−0.072
	(−1.468)	(−0.015)	(−1.775)	(1.781)	(−0.242)	(−0.596)
*Soe*	0.218 *	0.689 ***	0.435 ***	0.189 *	-	-
	(1.924)	(3.391)	(2.608)	(1.857)	-	-
*Mshare*	−0.029	0.061	−0.006	−0.068	−0.080	0.051
	(−0.621)	(0.850)	(−0.087)	(−0.985)	(−1.113)	(1.105)
*Top1*	−0.005	0.016 ***	−0.002	0.003	−0.001	0.004
	(−1.584)	(3.036)	(−0.562)	(0.630)	(−0.278)	(1.098)
*Board*	−0.119	0.138	−0.104	0.101	0.101	−0.007
	(−1.407)	(1.332)	(−0.759)	(1.156)	(0.837)	(−0.085)
*Dual*	−0.035	0.063	−0.033	0.115 *	−0.000	0.013
	(−0.442)	(0.988)	(−0.321)	(1.823)	(−0.002)	(0.224)
*Constant*	−0.868	0.019	2.311	3.696 ***	3.218	2.057 *
	(−0.921)	(0.012)	(0.771)	(2.677)	(1.212)	(1.685)
*Year Fixed Effects*	Yes	Yes	Yes	Yes	Yes	Yes
*Firm Fixed Effects*	Yes	Yes	Yes	Yes	Yes	Yes
*R-squared*	0.483	0.497	0.307	0.530	0.497	0.399
*Observations*	2303	2304	1970	2637	1565	3042

Note: t-statistics are reported in parentheses. ***, **, and * represent statistical significance at the 1%, 5%, and 10% levels, respectively.

**Table 7:** Mechanism analysis.

Variable	(1)	(2)
	*TFP*	Patent
*ESOP*	0.055 ***	0.016 **
	(3.082)	(2.092)
*Size*	−0.116 ***	−0.001
	(−5.070)	(−0.329)
*Lev*	0.105	0.004
	(1.361)	(0.242)
*Roa*	1.300 ***	−0.079 *
	(10.439)	(−1.727)
*Growth*	0.215 ***	0.002
	(10.903)	(0.366)
*Cash*	−0.027	0.048 *
	(−0.377)	(1.821)
*Tangibility*	0.279 ***	−0.001
	(3.358)	(−0.047)
*Age*	0.070 **	0.002
	(2.268)	(0.151)
*Soe*	−0.163 ***	−0.007
	(−3.023)	(−0.416)
*Mshare*	0.012	−0.006
	(0.665)	(−1.027)
*Top1*	0.001	0.000
	(0.425)	(0.225)
*Board*	0.018	−0.014
	(0.709)	(−1.340)
*Dual*	0.009	−0.006
	(0.629)	(−1.173)
*Constant*	5.683 ***	0.083
	(11.919)	(0.846)
*Year Fixed Effects*	Yes	Yes
*Firm Fixed Effects*	Yes	Yes
*R-squared*	0.838	0.167
*Observations*	4607	4607

Note: t-statistics are reported in parentheses. ***, **, and * represent statistical significance at the 1%, 5%, and 10% levels, respectively.

## Data Availability

The data presented in this study are available on request from the corresponding author.

## Appendix A

**Table A1:** The number of enterprises and employees by sector.

Sector	Enterprises in 2014	Employees in 2014	Enterprises in 2020	Employees in 2020
Mining	61	1,839,237	70	1,793,143
Textiles	25	1,350,34	32	153,757
Fur products	5	22,876	9	33,395
Paper making and paper products	18	69,338	25	109,956
Petroleum	11	59,408	15	59,529
Chemical	133	414,097	227	629,180
Pharmaceutical	140	471,910	218	745,300
Chemical fiber	18	75,575	22	166,587
Rubber and plastic	38	120,937	73	224,637
Black (non-ferrous) metal smelting and processing	83	848,259	99	794,632
Total	532	4,056,671	790	4,710,116

**Table A2:** The number of enterprises and employees by ownership.

Ownership	Enterprises in 2014	Employees in 2014	Enterprises in 2020	Employees in 2020
State-owned enterprises	221	3,185,762	236	3,036,109
Non-state-owned enterprises	311	870,909	554	1,674,007
Total	532	4,056,671	790	4,710,116

**Table A3:** The number of enterprises and employees by ESOP.

	ESOP Enterprises		Non-ESOP Enterprises	
Year	Enterprises	Employees	Enterprises	Employees
2014	8	29,970	524	4,026,701
2015	66	227,171	488	3,885,386
2016	33	136,567	560	4,079,442
2017	32	109,949	608	4,180,064
2018	30	200,432	712	4,398,799
2019	23	99,473	733	4,541,346
2020	42	267,252	748	4,442,864
Total	234	1,070,814	4373	29,554,602

## Appendix B

**Table A4:** Equilibrium hypothesis testing: propensity score matching.

Covariate	Sample	Mean Difference		*T*-Value (*p*-Value)	Standard Deviation (%)	
		Treatment Group	Control Group		Standard Deviation	Decrease (%)
*Size*	Before matching	22.222	22.212	0.27 (0.785)	0.8	
	After matching	22.222	22.184	0.98 (0.326)	3.2	−294.9
*Lev*	Before matching	0.398	0.410	−2.01 (0.045)	−5.8	
	After matching	0.398	0.398	−0.08 (0.934)	−0.3	95.1
*Roa*	Before matching	0.048	0.041	4.07 (0.000)	11.8	
	After matching	0.048	0.048	−0.11 (0.914)	−0.4	96.8
*Growth*	Before matching	0.196	0.146	4.67 (0.000)	13.2	
	After matching	0.195	0.209	−0.94 (0.347)	−3.7	71.8
*Cash*	Before matching	0.052	0.058	−3.15 (0.002)	−9.0	
	After matching	0.052	0.054	−0.67 (0.504)	−2.4	73.5
*PPE*	Before matching	0.253	0.295	−10.07 (0.000)	−29.5	
	After matching	0.253	0.251	0.35 (0.725)	1.2	96.0
*Age*	Before matching	2.157	2.222	−3.13 (0.002)	−9.1	
	After matching	2.157	2.178	−0.89 (0.375)	−3.0	67.1
*Soe*	Before matching	0.188	0.450	−19.57 (0.000)	−58.7	
	After matching	0.188	0.190	−0.13 (0.894)	−0.4	99.3
*Mshare*	Before matching	0.773	0.685	6.84 (0.002)	19.9	
	After matching	0.773	0.763	0.68 (0.498)	2.2	88.8
*Top1*	Before matching	33.955	35.520	−3.75 (0.000)	−11.1	
	After matching	33.930	33.491	0.91 (0.365)	3.1	72.0
*Board*	Before matching	2.196	2.234	−5.82 (0.000)	−16.6	
	After matching	2.196	2.200	−0.49 (0.623)	−1.7	89.7
*Dual*	Before matching	0.275	0.239	2.94 (0.003)	8.3	
	After matching	0.275	0.253	1.43 (0.152)	5.0	39.2
